# Salient Object Detection Based on Optimization of Feature Computation by Neutrosophic Set Theory

**DOI:** 10.3390/s23208348

**Published:** 2023-10-10

**Authors:** Sensen Song, Yue Li, Zhenhong Jia, Fei Shi

**Affiliations:** 1 Key Laboratory of Signal Detection and Processing, College of Computer Science and Technology, Xinjiang University, Urumqi 830046, China; song_sen_sen@163.com (S.S.); liyue_youxiang@163.com (Y.L.);; 2 College of Mathematics and System Science, Xinjiang University, Urumqi 830046, China

**Keywords:** salient object detection, feature optimization, neutrosophic set theory, low-rank matrix recovery model

## Abstract

In recent saliency detection research, too many or too few image features are used in the algorithm, and the processing of saliency map details is not satisfactory, resulting in significant degradation of the salient object detection result. To overcome the above deficiencies and achieve better object detection results, we propose a salient object detection method based on feature optimization by neutrosophic set (NS) theory in this paper. First, prior object knowledge is built using foreground and background models, which include pixel-wise and super-pixel cues. Simultaneously, the feature maps are selected and extracted for feature computation, allowing the object and background features of the image to be separated as much as possible. Second, the salient object is obtained by fusing the features decomposed by the low-rank matrix recovery model with the object prior knowledge. Finally, for salient object detection, we present a novel mathematical description of neutrosophic set theory. To reduce the uncertainty of the obtained saliency map and then obtain good saliency detection results, the new NS theory is proposed. Extensive experiments on five public datasets demonstrate that the results are competitive and superior to previous state-of-the-art methods.

## 1. Introduction

Regarding an image, we are only interested in the salient regions, as they are the most fascinating and expressive regions [1]. Due to the uniqueness of these features and the absence of prior knowledge, salient maps obtained through traditional theory-based algorithms [2,3,4,5,6,7,8,9,10,11,12,13,14] heavily rely on inherent features of both the object and the background in the image. These attributes encompass color, brightness, texture, position, and contrast [2,3,4,5,6,7,8], all of which pose challenges in isolating the object’s features. Hence, the greater the number of features fused, the easier it is to identify the object. However, this is performed without taking into account the algorithm’s structural merits and performance. Furthermore, in real-world application scenarios, an excess of fused low-level features may potentially lead to deteriorated saliency detection. We posit two primary reasons for this: first, as the number of features increases, so does the likelihood of redundant information, surpassing the algorithm’s processing capacity. This can result in the salient object information being overshadowed by extraneous features. Secondly, with an abundance of features, there is also the potential for non-objective features to appear more salient, resulting in a similar issue akin to missing feature information. In this paper, we have experimentally demonstrated the critical importance of selecting features that align with the algorithm’s structure, yet differ sufficiently for subsequent target feature processing.

**Figure 1:**
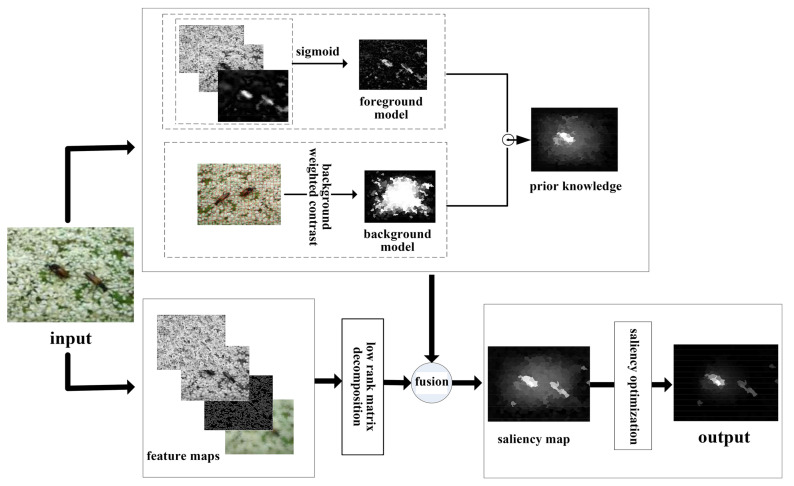
The structure of the proposed algorithm. The first step is to extract and select the input image features; one part is to construct prior knowledge, and the other part is applied as the input of low-rank matrix decomposition. The prior knowledge and output of low-rank decomposition are then fused to complete the salient object detection. The output is obtained by our saliency optimization algorithm.

Furthermore, the effective organization, discrimination, and rectification of the features of the object and background, along with the accurate classification of feature pixels, require an analysis of their characteristics and patterns. This process involves uncovering the inherent connections between features and subsequently constructing an algorithmic model. Low-rank matrix decomposition methods [15,16], rooted in an understanding of low-level features like image color, brightness, texture, position, and contrast, are well-suited for extracting object features. These methods operate on the assumption that an image can be partitioned into a low-rank section containing highly redundant information (e.g., visually consistent background region) and a sparse salient section (e.g., unique foreground target region). Consequently, the feature matrix of the input image can be deconstructed into a low-rank redundant matrix corresponding to the background and a sparse matrix corresponding to the salient object. After the features are decomposed, those within the sparse matrix represent the most refined expression of the image’s content, revealing its unique information. Nevertheless, it remains uncertain whether it encompasses the complete object information, or how to highlight the object information as prominently as possible. To address this issue, introducing prior knowledge of the object is a valuable approach. Adopting an iterative fusion approach to incorporate this prior information highlights the object information and completes the initial salient object detection. As shown in Figure 1, the initially obtained saliency map contains too much redundant and interfering information, which affects the results of saliency detection. Therefore, further optimization is necessary. Moreover, it is worth noting that the majority of saliency maps are now presented as hierarchical multi-valued images, where many region pixels have values within the range of [0,1], and the distinctions between these features are inherently fuzzy. This paper proposes that transforming the salient optimization problem into a fuzzy processing problem is an important approach. The NS theory [17,18] offers significant advantages in handling fuzzy problems. It extends the scope of fuzzy theory, broadening its applications and enabling a more precise expression and processing of fuzzy and uncertain information. Therefore, we present a salient object detection algorithm based on feature computation optimization using neutrosophic set theory (SMNS). The following is a list of our major contributions.

1. We show that the selection of features should be focused on their uniqueness rather than their quantity. Prior object knowledge is constructed using the foreground and background models, integrating pixel-wise and super-pixel cues. In this way, we effectively obtain the main information about the object.
2. We provide a new mathematical definition of neutrosophic set theory, redefining the formulas for its three subsets: True (T), Indeterminacy (I), and False (F).
3. A new fusion update method is applied to highlight object features and achieve salient object detection, significantly improving the saliency map’s overall quality and accuracy.

The rest of the paper is structured as follows: Section 2 presents related works. Section 3 describes the proposed salient object detection approach in detail. Section 4 presents experimental results in comparison to other approaches, as well as discussions. Section 5 concludes with conclusions.

## 2. Related Works

In most traditional theory-based saliency detection algorithms, low-level features, spatial information, and central prior knowledge are employed to generate the object information. In 1998, Itti et al. [19] proposed a central enveloping contrast, which detects salient objects by using color, intensity, and directional features. Huo et al. [20] exploited the link between several color attributes and their spatial information to describe an image’s global information. Borji and Itti [2] employed the sparsity of local and global image blocks as two complementing processes to design saliency models. Goferman et al. [3] assumed that salient objects have a compact spatial structure and subsequently detect salient objects based on the difference information of the image’s spatial structure. In [13], a new salient object detection method based on foreground, center, and background is proposed that employs regional color as the foreground and highlights salient objects by integrating perceptible uniform color differences within the region as the foreground. According to [14], the saliency map of an image can be produced via a global estimate of linear color combinations in a high-dimensional color space, and then the performance of saliency detection is further improved by additional local features and learning-based algorithms. Other works [4,5] believe that the salient object is generally framed toward the center of the image and that the saliency map is constructed using central prior knowledge. However, salient objects sometimes exist outside the image center in many images, resulting in a central prior mapping that improperly suppresses salient areas far from the image center while highlighting some background regions near the center.

In recent years, image boundaries have been routinely used as background prior information for salient object detection [5,6,7,8,9,10,11,12]. The saliency estimation problem is transformed into a rank-and-retrieve task in [10], with image boundary information taken as background prior knowledge. In [6], the difference between the object and the background is assumed to be on the shortest path to the image boundary. The separation strategy for the object and background is based on the very short distance between the background region and the boundary and the relatively long distance to the object. Zhu et al. [8] proposed the concept of background connectivity, which determines whether a region belongs to the background by evaluating the extent of connectedness between the region and the boundary. Yuan et al. introduced a saliency regression correction approach in [12], which improves the accuracy and robustness of saliency estimates based on prior boundary knowledge by correcting and deleting foreground super-pixels near the boundary. While edge priors can improve the accuracy of salient object detection, they still face many challenges, such as the processing of saliency map details not being satisfactory under low-level features. Therefore, we drew inspiration from related algorithms in the fields of deep learning [21,22], image segmentation [23], and image registration [24] to conduct research on salient object detection based on traditional theories.

## 3. Proposed Algorithm

In this section, we provide a detailed overview of the proposed algorithm, illustrated in Figure 1. The proposed algorithm comprises two main parts. The first part involves constructing the detection algorithm for salient objects. Initially, we establish prior object knowledge using foreground and background models that incorporate pixel-wise and super-pixel cues. Subsequently, we select and extract feature maps for computation. This step aims to effectively differentiate the object features from the background features in the image. Ultimately, we fuse the features decomposed by the low-rank matrix recovery model with the prior object knowledge to accomplish salient object detection. The second part is to optimize the saliency map. The results of salient detection preserve the mutual uncertainty between object and background information in the image. As a result, saliency optimization becomes a fuzzy processing issue. Given that NS theory has demonstrated advantages in addressing fuzzy problems, it proves to be well-suited for saliency optimization. Therefore, we propose a novel mathematical description of the NS theory to optimize the saliency map and improve object detection accuracy.

### 3.1. Saliency Object Detection

#### 3.1.1. Feature Extraction and Selection

In salient object detection algorithms, the low-level features of an image or depth features based on these inherent features are critical. Furthermore, feature selection is also important. The following are descriptions of the features chosen for this paper:

- *Color features*: Because the L*a*b* color space is based on human perception of color and includes all perceptible colors, its color gamut exceeds that of the red, green, and blue (RGB) color spaces, making it appropriate for image processing. Its three components, as well as its color volume, are tetra-chromatic features. The color volume is defined in [13] as:
  (1) $$cv_{Lab}\left(i,j\right)=4/3\pi \times L\left(i,j\right)\times a\left(i,j\right)\times b\left(i,j\right)$$
- *Image local information entropy*: Image entropy is a statistical form of feature that represents the average amount of information in an image. The local entropy of the image is defined according to the order of the gray distribution of the pixels, which reflects the richness of the image information. The greater the entropy, the clearer the image details [25,26].
- *Guided image filtering* [27]: As the name implies, a guided filter is required to have a guide to filter, and different guided images can be selected depending on their different functions. We chose to utilize it to extract edge features in this study, so its input is the image itself.
- *Gradient* [28]: Gradient images are images with directional variations in grayscale intensity or color. The mathematical definition of the gradient is a vector, indicating that the directional derivative of a function at that location takes the largest value along that direction. As a result, the image can be viewed as a two-dimensional discrete function, and its grayscale gradient is the derivation of this two-dimensional discrete function. The difference can be used instead of the differential to derive the image’s grayscale gradient. Then, we obtained different features by setting the gradient operator.

As previously stated, insufficient feature information prevents the object from being entirely highlighted, resulting in saliency map degradation and over-segmentation issues during the subsequent segmentation process. The more feature information that surpasses the algorithm’s processing capability, the more worthless information appears in the results, lowering the saliency map’s quality. Under-segmentation will undoubtedly occur in this case. Some features can be highlighted, resulting in the same problem with fewer features as before.

**Figure 2:**
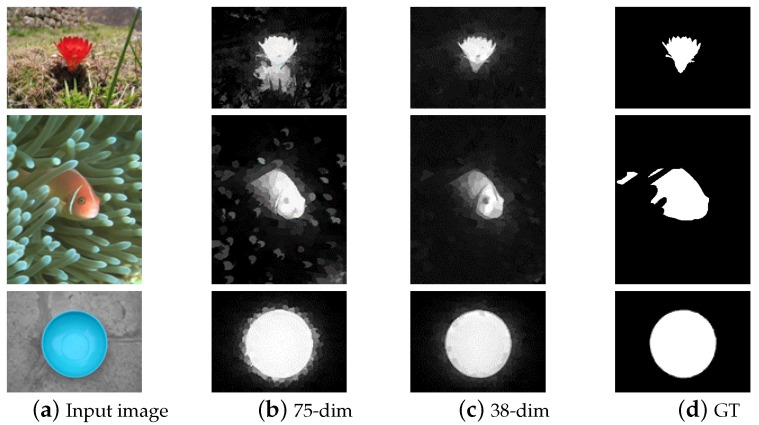
Comparison between the 75-dimensional feature saliency map in the second column and the 38-dimensional feature in the third column.

In [14], 75-dimensional feature vectors and 38-dimensional feature vectors were extracted from images for subsequent saliency map estimation. The experimental results are shown in Figure 2, demonstrating that having too many features is not always optimum for detecting salient objects.

**Figure 3:**
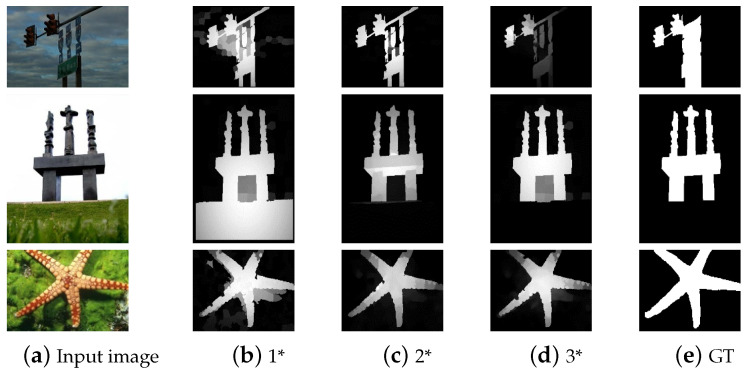
Comparison of salient object detection results of feature combinations 1*, 2*, and 3*.

In Table 1, the feature combinations are designated 1*, 2*, and 3*. The optimum detection effect may be obtained only if the feature set is appropriate for the proposed algorithm. The performance of our algorithm for the three sets of features is shown in Figure 3. These results demonstrate that the selection of features should not be based on quantity but on the rational combination of features and the analysis of the algorithm structure, which can remove redundancy and extract useful information. Therefore, the feature combinations 2* is employed as the input for subsequent feature computation in this paper, namely the feature maps illustrated in Figure 1.

#### 3.1.2. The Prior Object Knowledge

To obtain the best object saliency map, prior knowledge of the object, which will provide us with the most significant possible help, is indispensable. In this paper, prior knowledge based on the foreground and background models, incorporating pixel-wise and super-pixel-wise cues, is built. In [8], a background model with good robustness and accuracy is provided, and its structure is expressed as follows:

(2) $$Bg\left(p\right)=Ctr\left(p_{i}\right)*w_{i}^{bg}$$

where Ctr(p) and $w_{i}^{bg}$ represent the contrast of the *i*th super-pixel and its background probability, respectively, and $p_{i}$ is the *i*th super-pixel in the image. Ctr(p) is the sum of the contrasts of the *i*th super-pixel with other super-pixels. The probability $w_{i}^{bg}$ is the similarity between the *i*th super-pixel $p_{i}$ and the background.

The SLIC method is employed as an image super-pixel preprocessing algorithm in this work since it has fewer parameters and a faster performance when compared to other super-pixel techniques. However, a background model based on the SLIC method with erroneous super-pixel bounds may result in the loss of some object information. As a result, to address the shortcomings of the background model and gain complete object information, we propose a pixel-based foreground model, and its flowchart is as follows: first, the input image is transformed to the local entropy map G1 [31], guided image filtering map G2 [27], and the grayscale image G3. Salient feature map G2’ is obtained based on G2 by Equation (3) in [32].

(3) $$G2^{\prime }\left(x\right)=\left\|G\left(u\right)-G2\left(x\right)\right\|$$

where G(u) is the mean pixel value of G2.

Finally, G1, $G2^{\prime }$, and G3 are weighted and superimposed, and the object is highlighted by the sigmoid function to obtain a saliency map.

The foreground model is defined as follows:

(4) $$Fg\left(x\right)=1/\left(1+e^{-(G1+G2^{\prime }+G3)}\right)$$

Here, the foreground model mainly enriches the details of the image. Additionally, it also makes up for the deficiency of the background method because the background model considers the pixel area connecting the boundary as the background, which causes the object on the boundary to be regarded as the background. Therefore, we obtain relatively complete prior target knowledge by combining the foreground model and the background model. The specific fusion steps of the two models are described as follows: first, the common features of the foreground and background models can be generated by using the intersection operation, and its mathematical expression is presented as follows:

(5) $$P_{\left(Fg\cap Bg\right)}=\vartheta_{1}e^{\left(-(Fg+Bg)\right)}$$

where Fg and Bg denote the foreground model in Equation (4) and the background model in Equation (2), respectively, and $\vartheta_{1}$ is a normalization coefficient.

The difference method is applied to obtain different features of the foreground model and the background model. It is defined as follows:

(6) $$P_{\left(Fg-Bg\right)}=\vartheta_{4}|\vartheta_{2}e^{Fg}-\vartheta_{3}e^{Bg}|$$

where we set the difference coefficients $\vartheta_{2}$ = 1.2 and $\vartheta_{3}$ = 0.8. $\vartheta_{4}$ is a normalization coefficient.

Then, to further optimize the features of the salient object, the correlation matrix $C_{\left(Fg,Bg\right)}$ is obtained by calculating the correlation between the foreground model and the background model in a 9*9 region. The common features and different features are combined and multiplied by the correlation matrix to obtain the salient features of the object $P_{s}$.

$$C_{\left(Fg,Bg\right)}=\left[\begin{array}{c} \begin{array}{ccc} \rho_{1,1}\left(Fg_{\left(9\times 9\right)},Bg_{\left(9\times 9\right)}\right) & \cdots & \rho_{m,1}\left(Fg_{\left(9\times 9\right)},Bg_{\left(9\times 9\right)}\right)\\ \vdots & \ddots & \vdots \\ \rho_{1,n}\left(Fg_{\left(9\times 9\right)},Bg_{\left(9\times 9\right)}\right) & \cdots & \rho_{m,n}\left(Fg_{\left(9\times 9\right)},Bg_{\left(9\times 9\right)}\right) \end{array} \end{array}\right]$$

where ρ(Fg,Bg) is the Spearman correlation coefficient, which is used to measure the correlation between the foreground and background models [33]. finally, the prior object knowledge is obtained by the following definition:

(7) $$P_{s}=C_{\left(Fg,Bg\right)}*\left(P_{\left(Fg\cap Bg\right)}+P_{\left(Fg-Bg\right)}\right)$$

The prior object knowledge $P_{s}$ of the image provides the object feature information for the feature computation in the next section.

#### 3.1.3. Feature Computation

The foundation for salient object detection is effectively organizing, discriminating, and correcting the features of the object and background to produce precise feature separation. As a result, feature separation techniques are critical, and we discover that the low-rank matrix decomposition approach is well suited for feature separation under the assumption that images can be separated into low-rank features and sparse features. Then, we can achieve feature separation using the low-rank matrix *L* and sparse matrix *S* of the image. Therefore, in this paper, the low-rank matrix decomposition algorithm described in [16] is employed for feature computation.

(8) $$\min_{L,S}\Psi \left(L\right)+\alpha \Omega \left(S\right)+\beta \Theta \left(L,S\right)\;\;\;s.t.\;F=L+S$$

where Ψ(L) is a constraint on a low-rank feature space. Here, Ω(S) indicates the $l_{1}-norm$. Θ(L,S) is a regularization term on *L* and *S* such that the separation of their two features is possible. α and β are positive trade-off parameters.

**Figure 4:**
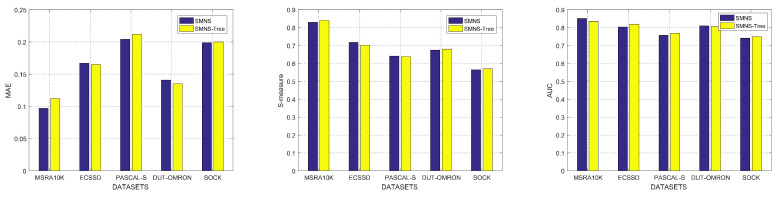
Comparison of low-rank matrix decomposition with or without tree structure in terms of MAE, S-measure, and AUC.

In terms of low-rank matrix decomposition, the most significant difference from [16] is that we remove the multilevel tree structure and change it to a single-layer structure, which accelerates the algorithm. Figure 4 shows that the performance of the low-rank matrix decomposition with a tree structure in paper [16] and our single-layer low-rank matrix decomposition in this paper are equivalent. However, the complex tree structure is removed in this paper, so the complexity of the algorithm is effectively reduced.

By performing the low-rank matrix decomposition algorithm using Equation (8), we obtain a sparse matrix *S* including salient object information and a low-rank matrix *L* containing the background portion.

#### 3.1.4. The Rule of Features Fusion

When the decomposition of the features is completed, sparse matrix *S* contains the most refined feature expression of the image content, representing the unique information in an image. However, it may not contain the desired object information. To solve this problem, the object prior knowledge $P_{s}$ provides the information of the object feature for matrix *S*, which can highlight the object features. Therefore, to further separate the object and background features, sparse matrix *S*, low-rank matrix *L*, and the prior object knowledge $P_{s}$ are combined to reduce the difference between *S* and $P_{s}$, and the effective object features are extracted. We define the following feature fusion update formula:

(9) $$\begin{array}{c} S^{\left(t+1\right)}=\left(I_{1}-\mu W^{-1}\right)\cdot S^{t}-\eta \cdot (diag\left(L\cdot \left(1-P_{s}\right)\right)+\\ diag\left(W\cdot S^{t}\right))*{\left(diag\left(P_{s}\cdot S^{t}\right)\right)}^{-1} \end{array}$$

where $I_{1}$ is the identity, $W=exp\left(\frac{{-w}_{i,j}}{\sigma^{2}}\right)$, $w_{i,j}={∥x_{i}-x_{j}∥}_{2}$ represents the weight between super-pixels, and $x_{i}$ and $x_{j}$ are the *i*th and *j*th pixel in the image. The parameters μ and η are the loss factor and the adjustment factor, which are set to 0.6 and 0.3, respectively. When *t* = 0, the initial value of S is obtained by Equation (8). $\left(I_{1}-\mu W^{-1}\right)\cdot S^{t}$ is the main body of object features with *t* iterations. $\left(I_{1}-\mu W^{-1}\right)$ is the object coefficient, which is used to highlight the object. $\eta \cdot (diag\left(L\cdot \left(1-P_{s}\right)\right)$ represents background features. $diag\left(W\cdot S^{t}\right))*{\left(diag\left(P_{s}\cdot S^{t}\right)\right)}^{-1}$ is the adjustment amount of the object features. By adding the prior knowledge, we can fine-tune the background feature and object feature by iteration, and separate the object and background as much as possible.

Although we have detected the object in the image, the saliency result still contains several blurs and noises. In the following section, we present an optimization approach based on NS theory that can remove the fuzzy value of the results and obtain a clean saliency map.

### 3.2. Saliency Optimization

The ultimate purpose of salient object detection is to accurately segment the object, which is a binary image that matches the ground truth. The majority of saliency maps are multivalued images with multiple regional pixels with values in the range [0, 1]. Because the distinction between these features is fuzzy, the saliency optimization issue can be turned into a fuzzy processing problem.

We discover that NS theory has a significant advantage in resolving uncertainty [34]. The basic idea of the NS theory is that all propositions and things have three memberships: True (T), Indeterminacy (I), and False (F), and their values are different. It is the handling of uncertainty that distinguishes it from other theories. In traditional sets, T and F are determined by thresholds; in fuzzy sets, membership functions are employed to determine whether they belong to T or F; and in NS, the values of the three components of T, I, and F are defined by different functions. In [35], the definitions of T, I, and F are as follows:

(10) $$T\left(i,j\right)=\frac{g\left(i,j\right)-g_{min}}{g_{max}-g_{min}}$$

(11) $$I\left(i,j\right)=\frac{G\left(i,j\right)-G_{min}}{G_{max}-G_{min}}$$

(12) $$F\left(i,j\right)=\frac{g_{max}-g\left(i,j\right)}{g_{max}-g_{min}}$$

where g(i,j) and G(i,j) are the intensity and gradient values at pixel coordinate (i,j), respectively.

As the NS theory is only a guiding theory (a set of principles), there are multiple mathematical descriptions of NS in different applications. Therefore, we introduce a novel mathematical description of the three subsets—T, I, and F—to deal with the fuzzy problem. We can observe that the variables in Equations (10)–(12) about the three subsets represent linear relationships, which makes it difficult to handle fuzzy information. For this reason, the ability of the three subsets to process the uncertainty can be increased by introducing nonlinear functions instead of linear functions. Moreover, we find that the sigmoid function is a classical nonlinear function that can be adjusted to obtain the T and F subsets by adjusting the parameter. For subset I, the idea of non-local mean filtering is utilized to reduce the uncertainty. Then, their definitions are as follows:

(13) $$T\left(x\right)=\frac{1}{1+exp\left(-\omega (x-\delta )\right)}$$

(14) $$I\left(x_{i}\right)=\frac{1}{C(x)}\sum_{\forall j}f\left(x_{i},x_{j}\right)g\left(x_{j}\right)$$

(15) $$F\left(x\right)=\frac{1}{1+exp\left(-\gamma (x-\zeta )\right)}$$

where ω, δ and γ, ζ control the steepness of the curve, and are set to 0.2, 0.5 and 0.1, 0.1.

$$C\left(x\right)=\sum_{j}exp\left(-∥x\left(N_{i}\right)-x\left(N_{j}\right){∥}_{2}^{2}/h^{2}\right)$$

and $N_{i}$ denote the number of neighbor pixels around pixel *i*, and *h* is the percentage of filter frequency.

$$f\left(x_{i},x_{j}\right)=exp\left(-∥x\left(N_{i}\right)-x\left(N_{j}\right){∥}_{2}^{2}/h^{2}\right)$$

represents the similarity between two points. $g\left(x_{j}\right)=P_{s}\left(x_{j}\right)*S\left(x_{j}\right)$ is the data to be processed. *x* is the pixel in the image. $x_{i}$ and $x_{j}$ is the *i*th and *j*th pixel in the image.

The process of saliency optimization is relatively simple and straightforward, using each of the three newly defined subsets to process the salient object detection results obtained from Equation (9). The three optimized results are then fused to obtain the final saliency detection results. From Equations (13) and (15), it can be seen that T(*x*) and F(*x*) are the functions of sigmoid transformation, and their parameters are set differently. By adjusting the parameters, we can enhance or weaken the feature points to highlight the object and separate the object and background as far as possible. Inspired by the non-local algorithm for image denoising [36], we redefine I($x_{i}$), which maintains the pixel surrounding features while removing the noise points in similar regions. After the three components are obtained, they are fused to obtain the ultimate features of different regions and generate the saliency map of object detection, which is defined in Equation (16). The last term in Equation (16) relates to continuous saliency values:

(16) $$Sm=\left(\varpi \left(I-F\left(S\right)\right)+T\left(S\right)\right)\cdot I\left(S\right)+\sum_{i,j}w_{i,j}{\left(S\left(x_{i}\right)-S\left(x_{j}\right)\right)}^{2}$$

where ϖ is set to 0.8, $w_{i,j}$ is as in Equation (9), and $S\left(x_{i}\right),S\left(x_{j}\right)$ are the *i*th and *j*th element of *S* in Equation (9).

The inconsistency of the parameter settings of T(*S*) and F(*S*) are applied to separate similar eigenvalues. In this way, we can distinguish similar values and enhance them differently, resulting in the separation of object and background features. I(*S*) is applied to address the value of the approximate feature position and reduce the impact of the prominent outliers or noises. The final fusion processing improves the overall quality and accuracy of the output salient object detection and the visual smoothness of the saliency map.

## 4. Experiments

We perform a series of experiments to completely verify the SMNS algorithm, analyzing the outputs from three perspectives: benchmark datasets, salient object detection methods, and evaluation metrics.

### 4.1. Experiment Fundamentals

#### 4.1.1. Benchmark Datasets

**Figure 5:**
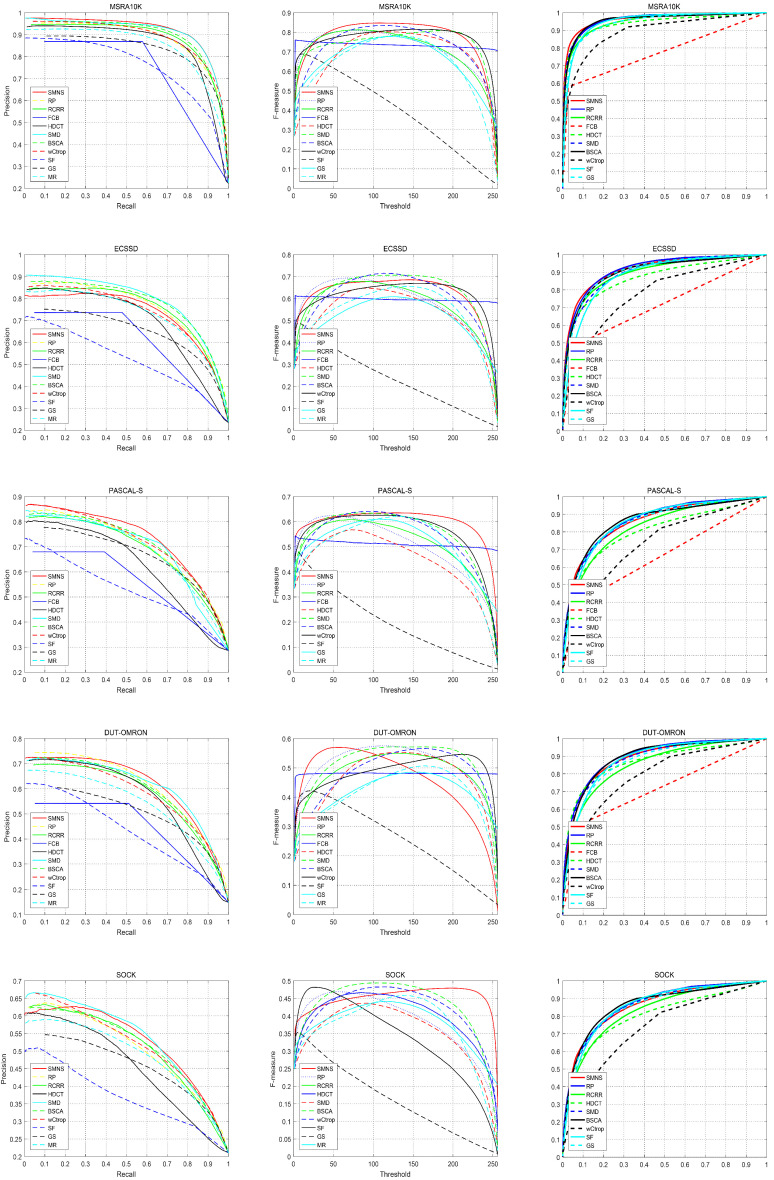
Comparison of five datasets in terms of P-R curves, F-measure, and ROC.

Five popular datasets are employed to validate our algorithm: MSRA10K [37], ECSSD [38], PASCAL-S [39], DUTOMRON [40], and SOCK [41]. There are 10,000 images in MSRA10K and 1000 in ECSSD, all of which contain simple scenes with obvious objects. PASCAL-S, DUT-OMRON, and SOCK are composed of images with relatively complex backgrounds and multiple salient objects, containing 850, 5168, and 1800 images of various sizes, respectively. Moreover, SOCK is obtained from SOC [41] by removing images of no salient object. From Figure 5 and Table 2, we can see that the performance of these evaluation metrics in these datasets is gradually degraded, which shows that the scenes contained in the images in these datasets are gradually more complicated.

#### 4.1.2. Salient Object Detection Methods

Our SMNS method is compared to current state-of-the-art traditional theory-based methods, namely SF [7], GS [6], wCtrop [8], MR [10], SMD [16], HDCT [14], BSCA [42], FCB [13], RCRR [12], and RP [43].

#### 4.1.3. Evaluation Metrics

To show the efficacy of the SMNS approach, the precision–recall (P-R) curve [44], F-measure curve [44], mean absolute error (MAE) [45], AUC [16], ROC [16], and S-measure [46] are employed. Precision (P) is the proportion of object pixels in the saliency map that are successfully detected compared to ground truth pixels, and recall (R) is defined as the ratio of correctly detected saliency object pixels to ground-truth object pixels. The F-measure is defined based on P and R with the following expressions:

(17) $$F=\frac{(1+\beta^{2})P\cdot R}{\beta^{2}P+R}$$

where $\beta^{2}$ is set to 0.3 to emphasize precision more than recall [44].

The P-R curve and F-measure are concerned with the proportion of correctly allocated salient pixels while ignoring detection results from non-salient area pixels. As a result, we introduce MAE to evaluate the accuracy of salient object detection.

(18) $$MAE=\frac{1}{M\times N}\sum_{k=0}^{M\times N}|S\left(k\right)-GT\left(k\right)|$$

The MAE is the pixel-wise average absolute difference between the saliency map S(k) and the ground truth GT(k).

The false positive rate (FPR) is represented by the ROC curve’s abscissa, while the true positive rate (TPR) is represented by the vertical coordinate, which corresponds to the true negative rate and false negative rate. The AUC is a metric used to compare the benefits and drawbacks of two categorization prediction algorithms.

(19) $$FPR=\frac{FP}{TN\times FP}$$

(20) $$TPR=\frac{TP}{TP\times FN}$$

where FPR and TPR denote the probability of being judged as positive but not positive and the probability of being judged as positive but also positive. FP is the false positive, TN is the true negative, TP is the true positive, and FN is the false negative.

The above evaluation metrics are all pixel-based similarity comparisons, without considering the similarity of the image structure. Therefore, the S-measure is applied to describe the structural similarity between the saliency map and ground truth, which is defined as follows [46]:

(21) $$\begin{array}{cc} S & =\alpha *S_{o}+\left(1-\alpha \right)*S_{r}\\ \; & =\alpha *(\mu *O_{FG}+\left(1-\mu \right)*O_{BG})+\\ \; & \left(1-\alpha \right)*\sum_{k=0}^{K}w_{k}*ssim\left(k\right) \end{array}$$

where α is in the range of [0,1] and μ is the proportion of the salient object to the ground truth. $S_{o}$ denotes an object-aware structural similarity measure, $S_{r}$ is the region-aware structural similarity measure, $O_{FG}$ is foreground comparison, $O_{BG}$ is background comparison, $w_{k}$ indicates the weight coefficient, and ssim(k) is a structural similarity measure.

#### 4.1.4. Parameter Settings

In this paper, we use source codes of the compared methods and data. The model parameters are set according to the referenced paper. Furthermore, the parameter settings in this paper are noted in the formulas, and the parameters are fixed for all experiments. We set the number of super-pixels to 250. All experiments are performed on a PC workstation with a 3.6 GHz CPU and 8 GB RAM using MATLAB 2016a.

### 4.2. Experimental Results and Analysis

#### 4.2.1. Performance of the SMNS Model with Some Feature Combinations

We demonstrate that the number of image features must be suitable for the proposed algorithm; otherwise, the salient detection results will be significantly worse.

**Figure 6:**
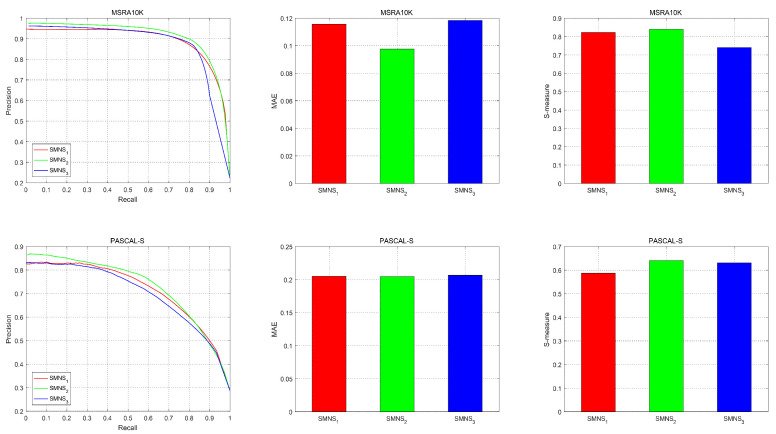
Comparison of the P-R curve, MAE, and S-measure results of datasets MSRA10K and PASCAL-S. SMNS ${}_{1}$, SMNS ${}_{2}$, and SMNS ${}_{3}$ indicate the results of feature combinations 1*, 2*, and 3*.

In MSRA10K, the feature combination 2* adopted in this paper is superior to the other two feature combinations concerning the three evaluation metrics. In PASCAL-S, the performance of the three combinations is comparable in terms of the MAE, but combination 2* performs best in terms of the S-measure and P-R curve. As revealed by the performance of the SMNS model with three feature combinations, which is shown in Figure 6, the selection of features depends not on the number of features but on their suitability for the algorithm.

#### 4.2.2. Comparison of Low-Rank Matrix Decomposition with or without Tree Structure

To fully evaluate the comparison of low-rank matrix decomposition with or without a tree structure, a series of experiments were carried out utilizing five benchmark datasets, including various scenarios and three evaluation metrics. Figure 4 shows that the performance of the low-rank matrix decomposition with a tree structure in the paper [16] and our single-layer, low-rank matrix decomposition in this paper are equivalent. However, the complex tree structure is removed, so the complexity of the algorithm is effectively reduced in theory. The complexity of the tree structure is O(M×N). When the tree structure is removed, the complexity becomes O(N), where *N* is the number of pixels and *M* is the number of root nodes of the tree. In addition, taking the average running time of each image in the dataset ECSSD as an example, the average time of an image with a tree structure is 1.524 s, while the average time without a tree structure is 1.205 s.

#### 4.2.3. Saliency Optimization of NS Theory

**Figure 7:**
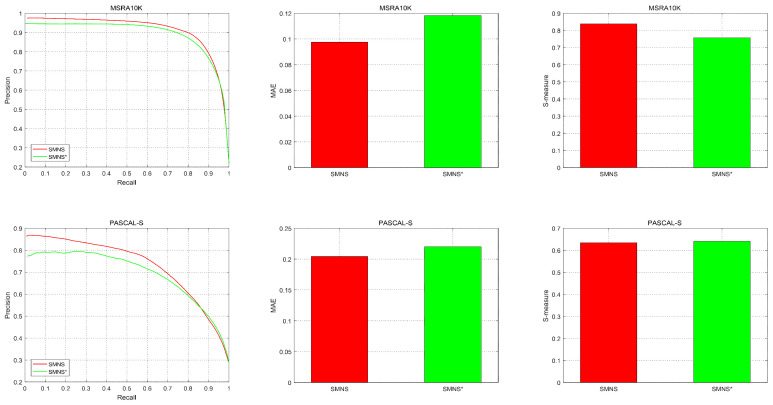
Comparison of the P-R curve, MAE, and S-measure results of datasets MSRA10K and PASCAL-S. SMNS indicates the result of the NS theory optimization process, and SMNS* has not been processed by NS theory.

To validate the effectiveness of the NS theory in optimizing the results, we conducted a comparative analysis on the saliency detection results with and without NS theory optimization, employing evaluation metrics such as P-R curves, MAE, and S-measure on both the MSRA10K and PASCAL-S datasets. This is illustrated in Figure 7. SMNS denotes the results optimized using the NS theory, while SMNS* represents results without NS theory processing. On the MSRA10K dataset, the performance of the three objective evaluation metrics for the results after NS theory optimization surpassed those without NS theory optimization. Moreover, the performance of the optimized results showed a significant improvement. On the PASCAL-S dataset, although the performance regarding the S-measure metric was comparable between the two, in terms of MAE and P-R curves, the results after NS theory optimization clearly outperformed those without NS theory processing. Through the comparative analysis above, it is demonstrated that the application of the NS theory in optimizing saliency detection results is highly effective.

#### 4.2.4. Validation of the Proposed Algorithm

**Figure 8:**
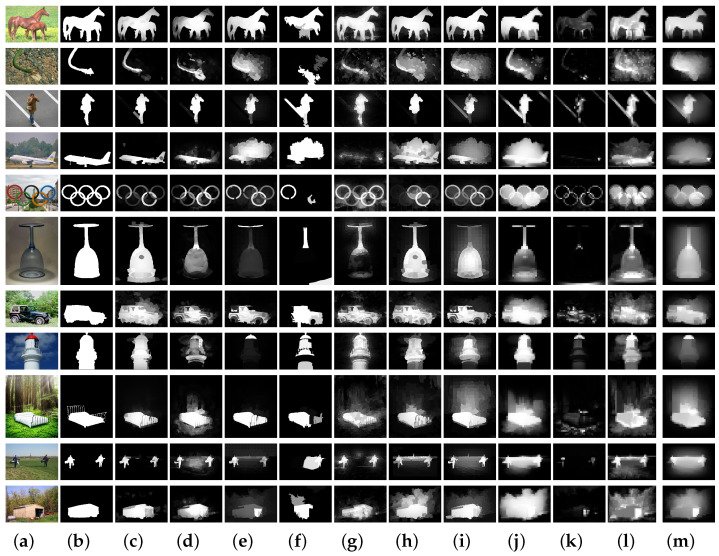
(**a**) Input; (**b**) GT; (**c**) SMNS; (**d**) RP; (**e**) RCRR; (**f**) FCB; (**g**) HDCT; (**h**) SMD; (**i**) BSCA; (**j**) wCtrop; (**k**) SF; (**l**) GS; (**m**) MR. Visual examples of different saliency detection methods on five datasets.

To completely assess the validity of our SMNS method, we ran a series of experiments with five benchmark datasets encompassing different scenes and six evaluation metrics, as well as ten state-of-the-art approaches for comparison. The proposed algorithm’s performance is depicted in Figure 5 and Table 2. In addition, Figure 8 shows some visual comparison examples of ten state-of-the-art approaches.

In Table 2, the proposed SMNS algorithm demonstrates an outstanding performance in terms of MAE. On three out of the five datasets, the evaluation metric MAE for the SMNS algorithm outperforms all the comparative algorithms, showcasing exceptional excellence. While on the PASCAL-S and DUT-OMRON datasets, the SMNS algorithm does not achieve the absolute best performance, it still ranks second, just behind the top-performing algorithms. Additionally, it is notable that the performance of evaluation metrics S-measure and AUC for the SMNS algorithm, while not as dazzling as MAE, is highly competitive. Specifically, the S-measure of this SMNS algorithm performs exceptionally well on all datasets except for SOCK. However, regarding the AUC performance, except for the MSRA10K and PASCAL-S datasets, where the SMNS algorithm performs well, on the ECSSD, DUT-OMRON, and SOCK datasets, its AUC performance is not as impressive as some comparative algorithms. This corresponds to the performance of the ROC evaluation metric in Figure 5 for the same reason, as it is determined by the nature of AUC.

In Figure 5, a comparison of all algorithm results across the five datasets is presented in terms of evaluation metrics, including P-R curves, F-measure, and ROC. It can be observed that on the MSRA10K, PASCAL-S, and DUT-OMRON datasets, the P-R curves enveloped by the SMNS algorithm are mostly superior to those of the comparative algorithms. Although it may not be the best on the other two datasets, the P-R curve envelopes of SMNS still exhibit a highly competitive performance. This indicates the accuracy of the SMNS algorithm in salient object detection. Since the F-measure is derived from the P-R curve, it can be inferred that the performance of the F-measure for the results of the SMNS algorithm is similar to that of the P-R curve. Additionally, across the five datasets, the performance of the ROC metric for the SMNS algorithm, while not the absolute best, is still among the top performers overall.

In conclusion, the results show that the proposed SMNS model can successfully address the aforementioned problems and increase salient object detection accuracy.

**Figure 9:**
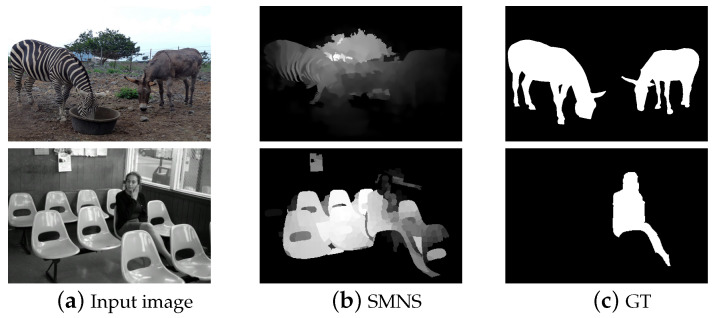
Some failure cases.

However, when researching salient object detection in complex scenes, we must also acknowledge the possibility of detection failures in practical applications. Figure 9 presents examples of salient object detection failures in complex scenes. In natural environments, objects may blend in with surrounding elements, making it challenging for the algorithm to accurately detect them. These cases contribute to a better understanding of the limitations of the algorithm and provide valuable reference for the improvement of subsequent algorithms.

#### 4.2.5. Parameters Analysis

In this study, several parameters are applied in the SMNS approach. Furthermore, the key parameters influence the performance of the final detection and classification results. Based on the content, structure, and correlation of the parameters in the study, these parameters are divided into four components. The first section contains the parameters for super-pixels; the second section discusses the fusion parameters for the foreground and background models; the third section provides the settings for low-rank matrix decomposition. The saliency optimization parameters are in the fourth section. Experiments using cross-validation are carried out to assess the effect of the parameters on the final detection and classification performance.

**Figure 10:**
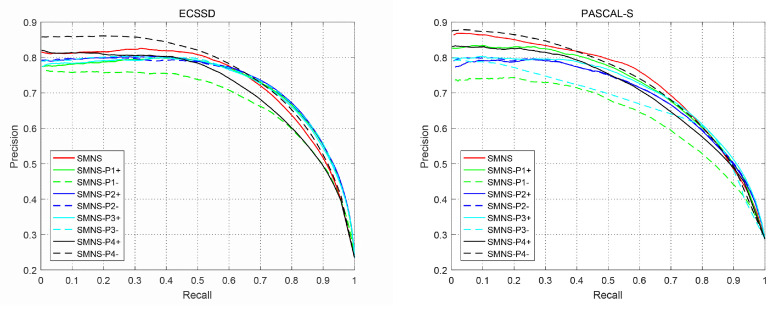
The impact of different parameters adjustments in terms of P-R curves, where SMNS-P1-4 represents four-part parameters. ‘+’ indicates that the parameters are adjusted in the direction of greater than the original value. ‘−’ indicates that the parameters are adjusted in the direction of less than the original value.

As shown in Figure 10, the performance of the P-R curve on the ECSSD and PASCAL-S is chosen as an example. We determine that the adjustment of all parameters impacts the results of the SMNS algorithm, and different parameters have different effects. Among them, the number of super-pixels has a significant influence on the SMNS algorithm. In contrast, the parameters of the second part have a lower impact. In addition, regardless of the direction of the second-part parameters adjustment, the performances are equivalent because these parameters only affect the prior knowledge and do not directly affect the salient object detection. The parameters of the third and fourth parts are also crucial to the SMNS algorithm of this paper. The former determines the extraction of object features. The latter controls the degree of optimization and can be employed to obtain better results than by changing other parameters. These parameters are critical, and the relationships among them are worth optimizing and exploring.

## 5. Conclusions

We present a novel salient object detection method based on the optimization of feature computation by NS theory. The prior object knowledge is provided for the fusion with the features obtained by feature computation, which can separate the object features and background features of the image as much as possible. The feature selected must be suitable for the algorithm. It is demonstrated that the NS theory, in which the formulas are redefined here, can be used as the optimization algorithm to obtain good saliency detection results. This approach for salient object detection offers some strategies. For instance, non-deep-learning algorithm features are introduced as prior knowledge for feature computation. A further extension of NS theory allows us to optimize the uncertainty in saliency maps. This methodological versatility and efficacy could find applications in a wide range of domains, including robotics, video analysis, and content creation. As biological brains are most effective in salient object detection, and as the NS theory is known in psychology, one of the research questions that can be addressed in the future is the development of brain-inspired techniques.

## Figures and Tables

**Table 1:** Three sets of image feature combinations.

Labels	Feature Descriptions	Dim
Color Features		
1*,3*	The average RGB values	3
1*,2*,3*	The average CIELab values	3
1*,3*	The average HSV values	3
1*,2*	The color volume of CIELab color space	1
Color Histogram Features		
1*	The RGB histogram	1
1*,2*	The CIELab histogram	1
1*	The hue histogram	1
1*	The saturation histogram	1
Color Contrast Features		
1*	The global contrast of the color features	9
1*	The local contrast of the color features	9
1*	The element distribution of the color features	9
Location Features		
1*,2*,3*	The average normalized x coordinates	1
1*,2*,3*	The average normalized y coordinates	1
Texture and Shape Features		
1*,2*	Area of super-pixel	1
1*	Histogram of gradients (HOG)	31
1*	Singular value feature	1
1*,2*	Gradients of image	1
Other Features		
1*,2*	Guided image filters	3
1*	Gabor filters [29]	48
1*	Steerable pyramids [30]	36
1*,2*	Local information entropy	1

**Table 2:** Comparison of five datasets in terms of MAE, S-measure, and AUC. The best three results are highlighted with red, blue, and green fonts, respectively.

Datasets	Evaluation	SMNS	RP	RCRR	FCB	HDCT	SMD	BSCA	wCtrop	SF	GS	MR
MSRA10K	MAE	0.097	0.121	0.112	0.120	0.143	0.104	0.125	0.108	0.179	0.155	0.156
	S-measure	0.830	0.781	0.791	0.719	0.797	0.839	0.813	0.824	0.553	0.772	0.771
	AUC	0.851	0.825	0.827	0.734	0.847	0.847	0.844	0.841	0.699	0.845	0.838
ECSSD	MAE	0.167	0.170	0.223	0.173	0.198	0.224	0.182	0.225	0.223	0.233	0.210
	S-measure	0.717	0.686	0.696	0.621	0.674	0.734	0.725	0.713	0.452	0.657	0.690
	AUC	0.804	0.785	0.795	0.684	0.785	0.812	0.815	0.798	0.603	0.793	0.810
PASCAL-S	MAE	0.204	0.204	0.212	0.213	0.229	0.206	0.222	0.201	0.237	0.241	0.243
	S-measure	0.641	0.583	0.600	0.515	0.568	0.633	0.634	0.650	0.489	0.618	0.626
	AUC	0.757	0.730	0.731	0.636	0.727	0.748	0.754	0.753	0.580	0.759	0.760
DUT-OMRON	MAE	0.141	0.139	0.182	0.149	0.158	0.164	0.191	0.145	0.151	0.210	0.218
	S-measure	0.674	0.672	0.660	0.605	0.656	0.670	0.652	0.647	0.513	0.620	0.614
	AUC	0.810	0.802	0.780	0.697	0.815	0.809	0.808	0.814	0.640	0.816	0.804
SOCK	MAE	0.199	0.224	0.242	-	0.234	0.223	0.245	0.238	0.214	0.259	0.276
	S-measure	0.565	0.564	0.582	-	0.553	0.599	0.588	0.586	0.434	0.564	0.569
	AUC	0.741	0.718	0.722	-	0.718	0.740	0.747	0.754	0.575	0.750	0.755

## Data Availability

The datasets generated and analyzed during this study are available in the mmcheng repository: https://mmcheng.net/code-data. These datasets were derived from the following public domain resources: http://mmcheng.net/msra10k. http://www.cse.cuhk.edu.hk/leojia/projects/hsaliency/dataset.html. http://cbi.gatech.edu/salobj. http://saliencydetection.net/dut-omron. https://mmcheng.net/code-data.
